# Channel Selection Based on Phase Measurement in P300-Based Brain-Computer Interface

**DOI:** 10.1371/journal.pone.0060608

**Published:** 2013-04-11

**Authors:** Minpeng Xu, Hongzhi Qi, Lan Ma, Changcheng Sun, Lixin Zhang, Baikun Wan, Tao Yin, Dong Ming

**Affiliations:** 1 Department of Biomedical Engineering, Tianjin University, Tianjin, China; 2 Institute of Biomedical Engineering, Chinese Academy of Medical Science & Peking Union Medical College, Beijing, China; UC Davis School of Medicine, United States of America

## Abstract

Most EEG-based brain-computer interface (BCI) paradigms include specific electrode positions. As the structures and activities of the brain vary with each individual, contributing channels should be chosen based on original records of BCIs. Phase measurement is an important approach in EEG analyses, but seldom used for channel selections. In this paper, the phase locking and concentrating value-based recursive feature elimination approach (PLCV-RFE) is proposed to produce robust-EEG channel selections in a P300 speller. The PLCV-RFE, deriving from the phase resetting mechanism, measures the phase relation between EEGs and ranks channels by the recursive strategy. Data recorded from 32 electrodes on 9 subjects are used to evaluate the proposed method. The results show that the PLCV-RFE substantially reduces channel sets and improves recognition accuracies significantly. Moreover, compared with other state-of-the-art feature selection methods (SSNRSF and SVM-RFE), the PLCV-RFE achieves better performance. Thus the phase measurement is available in the channel selection of BCI and it may be an evidence to indirectly support that phase resetting is at least one reason for ERP generations.

## Introduction

Brain-Computer Interfaces (BCIs) are communication systems that allow people to send information to a computer or commands to other electronic devices only by measuring brain activities without any body movement. Such systems can be considered as the solely way of communication for people who suffer severe neuromuscular diseases and are incapable of any motor functions but are cognitively intact [1],[2]. To date, in non-invasive functional brain monitoring methods, the electroencephalography (EEG) provides a preferable solution in most circumstances with a high time resolution as well as simple and affordable recording requirement [3].

The P300 speller is one of the most popular EEG-based BCI paradigms and provides many clinical applications [4]–[6]. As described by Farwell and Donchin, a P300 speller presents a character matrix on a computer display in front of BCI users. Each cell of the matrix contains a character and either row or column is intensified individually and randomly. Spelling with the BCI, users should pay attention to the character they wish to communicate with [7]. Since the occurrence rate of the row (column) containing the focused character is often below 20%, intensifications of this row (column) exert target stimuli and elicit P300 responses. The BCI system identifies these P300 potentials and transforms users’ attention to character output (i.e. the intersection of the row/column targets). The P300 response is an internal mechanism of the human brain, which allows the P300 speller to require no BCI user training [8].

The performance of the P300 speller depends greatly on the quality and amount of the information acquired from EEG records [9]. Nowadays, a great challenge of the P300 speller is the optimization of the number of electrodes for each user [9],[10]. A reduced number of electrodes will take less time to install, be more user-friendly, reduce the expense of BCI equipment and consume less power. This may efficiently support wireless EEG caps [10],[11]. Previous works on this subject pay most attention to neuroscience evidences. According to what the neurophysiology suggests, early research focused on the standard locations (i.e., Fz, Cz, Pz) [8],[12],[13]. Some offline studies suggest that the use of additional locations, particularly posterior sites, may improve classification accuracy [14]–[18], and a six/eight-electrode configuration is proposed to provide a satisfactory classification with appropriate use [19]–[22]. However, P300 signals are subject-specific [19], and the optimal EEG recording locations for P300 identification may vary in practice. It is possible that a different montage would be required for patients with various neuromuscular pathologies. Accordingly, an adaptive channel optimization method is necessary for practical applications to identify an individual montage [23].

The classical greedy strategy, known as ‘backward elimination’, has been popularly recommended and used in recent BCI channel selections [9],[10],[23],[24]. In general, it starts with a full set of electrodes according to the 10–20 system (covering all areas of the head) and reduces the number of required EEG channels while keeping the classification accuracy optimal [9]. A robust feature space should contain more identifiable but less redundant information. To this end, features worthless for accuracy should be removed. One approach is to use pure mathematical evidence, which is called dependent criteria, i.e., constructing a series of feature combinations and selecting the best one with the highest classification rate. Some methods in this approach, such as genetic algorithm or SVM-RFE, have been used successfully for channel optimization in BCIs [23]–[25]. However, it’s difficult to use them in P300 spellers because of the high computational complexity. Another approach, called independent criteria, is computationally simple. It directly evaluates all features, and then removes the identified “worthless” features. Algorithms of this method are popular in many fields, but rarely used in EEG feature selections. The comprehensive work [10] on this subject by Cecotti *et al* indicates that the cost function based on signal to signal-plus-noise ratio (SSNR) is better than that based on classification accuracy in terms of channel selection using backward elimination in P300 spellers. Meanwhile, a pre-processing using a spatial filter (SF) based on the xDAWN algorithm [26],[27] helps to select the optimal channels remarkably [10]. Thus it is promising to evaluate the importance of EEG features on the classification by using a global measure of EEG signals [10].

Up to now, there have been two distinct mechanisms to generate averaged ERP responses. The evoked model, as a basis of SSNRSF (i.e. SSNR plus SF), considers that the ERP response results from a superimposed neuronal activity with fixed-polarity and fixed-latency to background electroencephalographic oscillations [28]–[31], while the oscillatory model believes it is generated by a partial phase synchronization of the ongoing EEG [32]–[37]. The debate between the two models has existed for a long time, since neither of them can explain the evoked potential exclusively. However, some recent literature suggests that the event-related potential is at least influenced by oscillatory brain activity [38]–[40]. Phase relations reflect the cooperative interactions between anatomically disparate neural populations [41],[42]. Such cooperative brain processes that are detectable at various spatial scales are supposed to be fundamental to the dynamic organization of sensory and cognitive brain functions [43],[44]. In ERP studies, phase-based measurement provides robust and sensitive monitoring on task-related fluctuations [39]. Moreover, recent studies imply that it’s possible to evaluate how EEG features contribute to classification by using phase related measurement. On one hand, EEG records from the same or connected functional areas should show more phase synchronization than those from different or unconnected functional areas [43]. Such phase synchronization between two specific channels would also be different from target mental task to non-target [41]. On the other hand, task-relevant cortical areas make a great contribution to a mental task, and the corresponding channels should play an important role in classification. Therefore, channel selection through phase measurements is a promising approach in the P300 speller, but has never been reported.

In this study, different from additive evoked model based methods, the PLCV-RFE as a phase measurement based method is developed and tested to verify its effectiveness. By measuring phase relationship between EEG channels, the PLCV-RFE separates channels into diverse clusters, and then ranks them to ensure that the first *n* channels reflect as many important sources as possible.

This paper is organized as follows. Section 2 addresses the methodology including the BCI experiment and the PLCV-RFE algorithm. Section 3 presents the results and discussion.

## Materials and Methods

### 1. Ethics Statement

This study was approved by the Institution Research Ethics Board at Tianjin University. All subjects gave written informed consent after the nature and possible consequences of the study were explained.

### 2. Experiment

**Figure 1:**
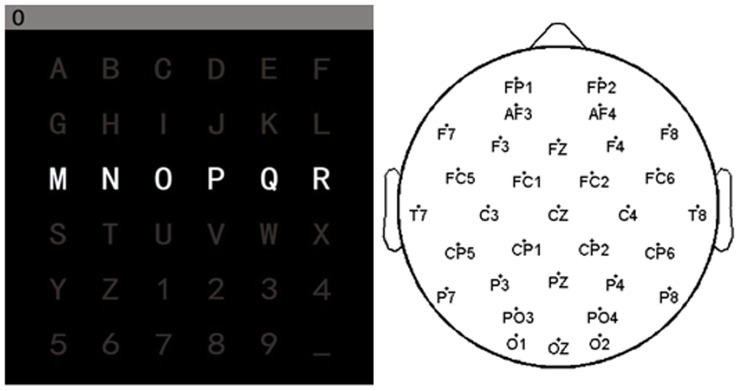
Character matrix and electrode locations. Left: 6*6 character matrix displayed in front of the participant with the third row intensifying. Right: 32-electrode locations follow 10–20 system in P300 spelling experiment.

The experiment paradigm followed Farwell and Donchin [7]. During the experiment, the participant sat in front of a computer monitor and viewed a 6 by 6 letter matrix (Figure 1). The task was to pay attention to a specified letter on the matrix and silently count the number of times the target character intensified, until a new character was specified for next selection. The character currently specified for selection was presented on the top left of the screen. At the beginning of each character block the matrix was blank for 2.5 s. Then, the rows and columns were intensified for 100 ms with 75 ms blank between intensifications. The matrix flashing presented 12 different stimuli to users and two of them contained the target character (one particular row and one particular column). A complete cycle of six row and six column intensifications constituted an epoch, and 15 epochs constituted a character block. 80 character blocks were conducted in the experiment for each subject. Thus there were 2,400 target trials and 12,000 non-target trials in this study.

Nine right-handed healthy subjects (23–25 years of age; 2 females) participated in the study. All subjects had no experience with a P300-based BCI system. The EEG signal was recorded by using a Neuroscan Synamps2 system with an EEG cap whose located electrodes follow the 10–20 system (Figure 1). All channels were referenced to the central lobe and grounded prefrontal lobe, and then re-referenced to the bilateral mastoid. EEG signals were bandpass filtered at 0.1–200 Hz, digitized at a rate of 1,000 Hz and stored. In the pre-processing EEG signals were first filtered to 0.1–40 Hz and downsampled at 200 Hz for phase measurement, and then downsampled at 20 Hz for classification. 700 ms of EEG after stimulus onset from the 32 channels was defined as the stimulus response and extracted. The first 40 character blocks were used for training, while the others were the test session.

### 3. Phase Locking and Concentrating Estimation

Phase locking values (PLVs) may be an effective approach to measure the variability of phase difference between two EEG signals [45]. However, neglecting the initial phase value makes it impossible to measure the degree of phase concentration which induces the ERP from the view of the oscillatory model. In this paper, we propose a novel method to estimate the phase locking and concentrating value (PLCV) of EEG signals.

The instantaneous phase can be obtained by the analytic signal. For casual signal 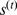, the analytic signal 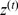 is a complex function defined as (1) and (2), where 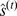 is the Hilbert transform of 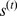.
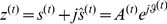
(1)Where *j* is the imaginary unit, *A* is the amplitude and *e* is the natural exponent.



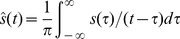
(2)If *x* and *y* denote the *x*th and the *y*th EEG channels, the phase locking value (PLV) should be defined as follows [45]: 
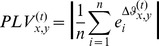
(3)where 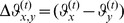,
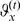
and 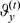 represent the instantaneous phase of EEG signals in the *x*th and the *y*th channels. The PLV is an average of *n* trials and ranges from 0 to 1. If channel *x* and channel *y* are more/less likely to be homologous, 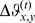 will show less/more variability, which brings a high/low PLV.

The phase concentration value can be defined similarly as follows: 
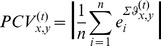
(4)where 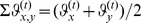. PCV derives from the idea of inter-trial phase coherence (ITC) whose equation is



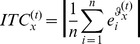
(5)It extends ITC to the condition of two channels. Thus PCV measures the consistency across the trials of two channels. If 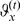 or 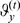 is phase-synchronized to a certain mental task, 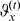 or 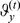 will be more constant when the task occurs according to the oscillatory model. Otherwise, 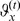 or 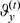 will be more random as it has no relation to the task. Therefore if channel *x* and channel *y* are more/less likely to be phase-synchronized to a certain mental task, 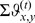 will show more/less concentration, which results in a high/less PCV.

PLV and PCV characterize different relationships between EEG behaviors of two locations. PLV measures whether they come from the same source, while PCV represents whether or not they are related to a mental task. Here we combine them to analyze ERPs and the phase locking and concentration value (PLCV) is defined as: 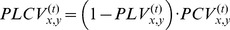
(6)



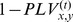
 is used here because homologous features rarely provide useful information to the recognition and they can be considered as having negative contributions. From (6) a high PLCV represents a pair of heterologous channels synchronized to a certain mental task.

### 4. Channel Ranking Using PLCV

It is the variance between target response and nontarget response that is the most important element in the recognition of event-related potentials. Then, the target effect of phase locking and concentration is defined as: 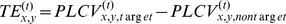
(7)


The TE value is a combined effect of mental task relativity and channel homology factors. A high value of 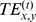 signifies that the phase concentration of channel *x* and channel *y* in target response is significantly higher than that in nontarget response, and such concentration is more likely to be multisource. However, a low 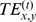 value may be a result of a homologous source, or the brain fluctuation behaving with little relation to the target mental task. For example, the value of 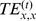 is zero for the same source reflected by channel *x* and itself. In this view, a low 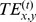 value may show that the EEG features are similar in the *x*th and the *y*th channels. Under this condition, if the features from the *x*th channel have been selected in the training of the learning machine, the *y*th channel will provide little additional useful information, and then can be regarded as a redundant channel, i.e., the *y*th channel contributes little to the classification.

**Figure 2:**
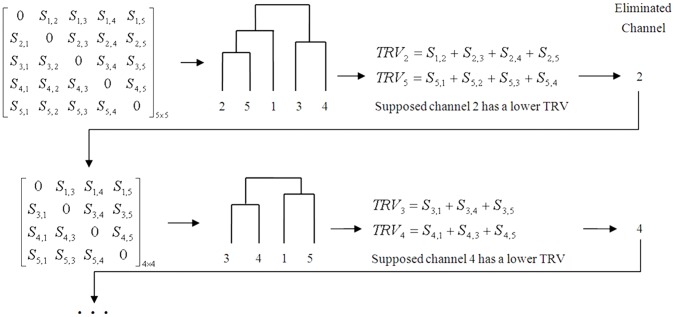
The computational process of RFE. A flow chart of RFE is shown with a 5-channel set. An earlier eliminated channel ranks lower.

However, it is hard to select the channel of the least contribution directly by evaluating TE measurements. To solve this problem, we use a hierarchy clustering method combined with a recursive strategy to group channels and iteratively rank each of them. We call this method “phase locking and concentrating value based recursive feature elimination (PLCV-RFE)”. In the clustering of PLCV-RFE, the TE is used to characterize the behavioral similarity between two channels. Since TE is a time varied value, we define the maximum value in the time window after the stimulus onset as the similarity,
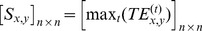
(8)where *n* is the number of channels. Once the *x*th and the *y*th channels are identified as the most similar couple among all available channel couples, the less important channel is identified with the lower task relativity value (TRV) which is defined as: .
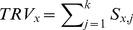
(9)where k is a decreasing variable in the recursive procedure. Its initial value is n. In each step of the recursive procedure, the current similarity matrix 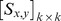 is used to construct a hierarchy cluster, in which each EEG channel is considered as a leaf on the hierarchy cluster tree Z. According to the Z, the couple with the least 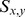 is identified at first, and then the channel with a lower TRV is eliminated from the identified couple. Before the next repetition, the similarity matrix needs to be reconstructed, since the number of channels surviving has been decreased. This process is carried out iteratively and all channels are sorted with ascending importance. Figure 2 illustrates a flow chart of PLCV-RFE that takes a 5-channel set as an example.

The Pseudocode of PLCV-RFE is as follows:

Initialize:

1. subset of surviving channels: *SC* = [1,2,…,32].
2. the size of *SC*: *k* = 32.
3. channel rank list: *R* = [].

Procedure:

1. calculate [*S_x,y_*]*_k_*_×*k*_, where *x,y*∈*SC*.
2. construct a hierarchy cluster tree Z by [*S_x,y_*]*_k_*_×*k*_.
3. group channels into *k*−1 classes by using the cluster tree Z.
4. identify the only class that contains two channels: *C* = [*p,q*], where *p,q*∈*SC*.
5. compute the TRV of each channel in *C*.
6. find the channel with lower TRV in *C*: *w* = argmin*_x_*(*TRV_x_,x*∈*C*).
7. update the feature ranked list: *R* = [*SC(w),R*].
8. reset *SC* = [*SC(1),*…,*SC(w*−*1),SC(w+1),*…,*SC(32)*], *k* = size(*SC*) and *C* = [].
9. repeat this procedure until *SC* = [].

*Output*: channel ranked list *R*.

### 5. Recognition Method

To evaluate the performance on a selected subset of channels, we measured the character recognition accuracy. 80-character spelling data for each subject was divided into two parts. The channel ranking ran on the first 40-character data, while the following 40-character data were used to calculate accuracies of the channel sets selected previously. Fisher’s Linear Discriminant Analysis (FLDA) was used for character recognition. As a benchmark method for BCI classification, FLDA has been proven to be capable of providing good performance P300-based BCI spelling [22].

## Results and Discussion

**Figure 3:**
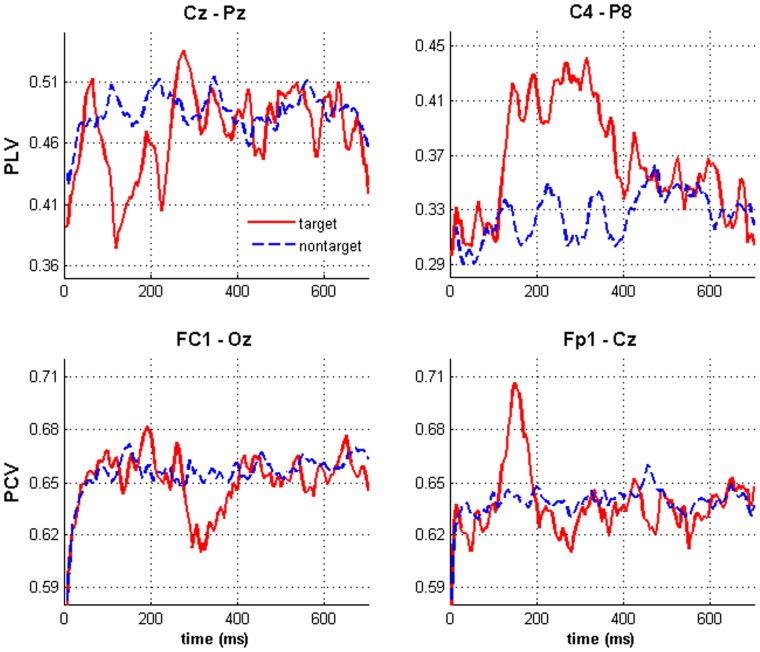
PLV and PCV presentations. Typical variations of PLV and PCV from target to nontarget are illustrated by certain channel pairs.

Figure 3 shows PLV and PCV differences between target and nontarget responses in certain channel pairs. In pair Cz and Pz, target PLVs achieve lower values from about 100 ms to 250 ms, while maintaining the level of nontarget at other times. In contrast, pair C4 and P8 gives higher target PLVs from about 100 ms to 450 ms. For PCV, the decrease and increase between nontarget and target can be found in pair Fc1-Oz and Fp1-Cz, respectively. Therefore, the phase measurement can reflect the changing relationship between channels.

**Figure 4:**
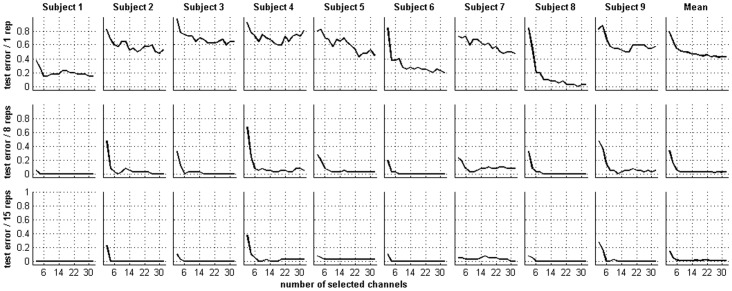
Character recognition errors of all subjects. CREs against the number of selected channels is shown in 1, 8 and 15 repetitions with all subjects.

Figure 4 presents the character recognition errors (CREs) against the number of selected channels by PLCV-RFE in 1, 8 and 15 epochs (repetitions). In most cases, the test error curves often increase smoothly or remain steady after a rapid decrease, with the number of selected channels increasing. In general, the more repetitions used, the fewer test errors. For a further analysis, the optimal channel subset (OCS) is proposed in this study, which results in the least test error with the least number of channels. For example, if 0%, the least CRE is achieved when 10 and 12 channels are used; then the OCS is the first 10 channels. Compared with the full channel set, the OCS has fewer CREs with fewer repetitions. For example, as illustrated by Table 1, the averaged CRE decreases 6.4%, 3.6%, 2.5%, 2.8%, 1.9% and 1.9% with 1, 2, 3, 4, 5 and 6 repetitions respectively. Six paired t-tests show that such decreases are significant (1 repetition: *t*(8) = −3.5, *p*-value<0.01; 2 repetitions: *t*(8) = −4.3, *p*-value<0.01, 3 repetitions: *t*(8) = −2.3, *p*-value<0.05, 4 repetitions: *t*(8) = −2.2, *p*-value<0.05, 5 repetitions: *t*(8) = −3.5, *p*-value<0.01 and 6 repetitions: *t*(8) = −2.2, *p*-value<0.05). In addition, with the increased repetitions, the size of OCS will be reduced significantly. For example, an average of less than 10 channels will be achieved in the OCS when using more than 6 repetitions.

**Table 1:** CRE comparison.

Repetitions	OCS size	CRE of OCS (%)	CRE of full channels (%)
1	22.1	36.4	42.8
2	20.6	19.4	23.1
3	17.4	12.2	14.7
4	17.2	5.3	8.1
5	17.4	2.5	4.4
6	13.4	2.2	4.2
7	9.8	1.7	2.5
8	7.9	0.8	2.2
9	8.1	0.8	1.4
10	9.9	0.3	0.6
11	8.2	0.3	0.6
12	8.0	0.3	0.6
13	5.2	0.6	0.8
14	5.2	0.6	0.6
15	7.3	0.3	0.6

**Figure 5:**
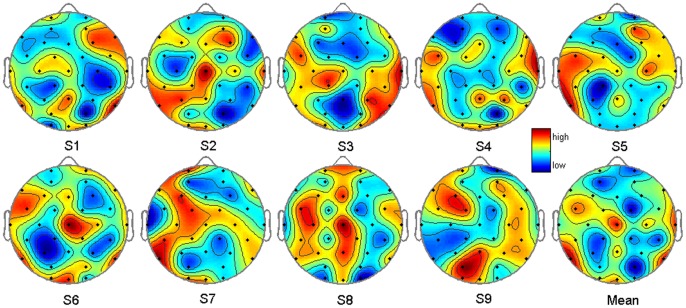
Channel weights of PLCV-RFE. Channel weights acquired by PLCV-RFE are displayed by the topography with all subjects and the averaged one.

The important channel sites may give a further understanding of the P300 speller. Table 2 illustrates the top 12 channel rankings of all subjects. P7, P8 and Cz are common to most of the subjects. In this study, all channels are weighted by z scores. P7 is the highest and wins, then followed by P8, Cz, T8, FC5, Oz and Pz. Figure 5 displays the channel weights by means of topography. The weight distribution is subject-specific. For example, the middle channels get less importance in S5, but they are essential to S8 to get a good classification performance. In the last averaged topography, the color distribution is approximately symmetric. Coherently with the neurophysiologists, Cz, Pz and Oz play important roles in the P300 speller. But on the contrary, Fz ranks 22^nd^, which shows less essential than FC5, the lateral channel in the frontal area. In addition, P7, P8 and T8 also contribute significantly to character recognition accuracy.

**Table 2:** List of channel ranks.

rank	s1	S2	S3	S4	S5	S6	S7	S8	S9	mean
*1*	P8	Cz	T8	T8	P7	Cz	P7	Cz	PO3	P7
*2*	F4	F4	FC5	FC5	FC5	FC5	F3	FC5	FC1	P8
*3*	F8	T8	P4	P4	T7	P8	CP5	Pz	F3	Cz
*4*	Pz	P7	CP1	P7	AF4	P7	FP1	C3	O1	T8
*5*	P7	P3	P8	Pz	C4	Oz	FC1	Fz	Pz	FC5
*6*	O2	CP1	C3	O2	P8	CP2	Oz	P3	AF4	Oz
*7*	FC6	C4	O1	P8	C3	C4	P8	FP2	F7	Pz
*8*	FC1	AF4	CP5	F8	FP1	FP2	CP1	FC6	P8	C4
*9*	AF4	Fz	O2	CP5	Pz	PO4	C3	P8	C4	F8
*10*	Cz	O1	C4	FC1	Cz	T7	CP6	P7	FP1	FC1
*11*	T8	F3	F7	Fz	FC1	O1	F7	T8	Oz	CP5
*12*	Oz	F7	P7	Oz	F4	F8	Cz	CP5	FC6	O1

**Figure 6:**
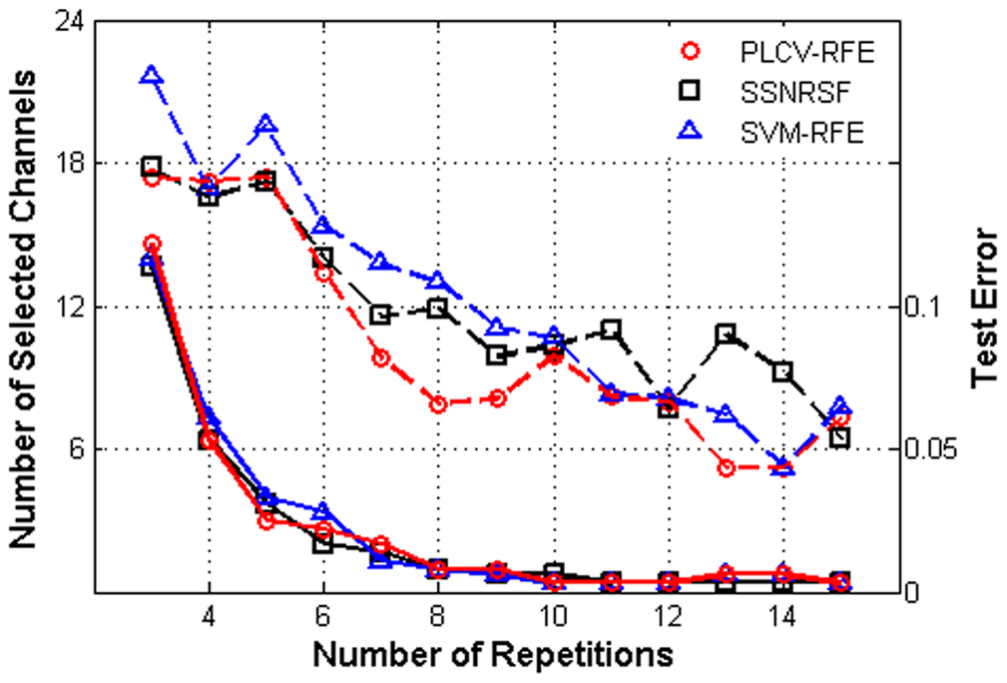
The comparison of optimal channel subsets. The number of OCS channels against the number of repetitions is displayed by dash lines with different methods. The corresponding CREs are illustrated by solid lines.

To confirm the efficiency of PLCV-RFE, two state-of-the-art feature selection methods, (SVM-RFE [24] and SSNRSF [10]), are involved in this study. Figure 6 shows the averaged size of OCS against the number of repetitions with corresponding CREs. The PLCV-RFE chooses fewer channels than SSNRSF and SVM-RFE in most cases. For example, in 8 repetitions, the OCS has 7.9, 11.9 and 13 channels for PLCV-RFE, SSNRSF and SVM-RFE respectively. Two paired t-tests prove that the PLCV-RFE is significantly superior to the other two methods in channel reduction (PLCV-RFE versus SSNRSF: *t*(134) = −1.9, *p*-value<0.05 and PLCV-RFE versus SVM-RFE: *t*(134) = −2.4, *p*-value<0.01). In addition, all three methods have comparable performance in CREs. For example, the least averaged CREs in 5 repetitions are 2.5%, 3.1% and 3.3% when using PLCV-RFE, SSNRSF and SVM-RFE respectively, and all CREs are 0.8% in 8 repetitions. Two-way analysis of variance (ANOVA) shows no significant difference among these methods in terms of CREs (*F*(2, 360) = 0.03, *p*-value = 0.97). Therefore, PLCV-RFE achieves a better performance, considering its OCS gets fewer electrodes without loss of accuracy.

**Figure 7:**
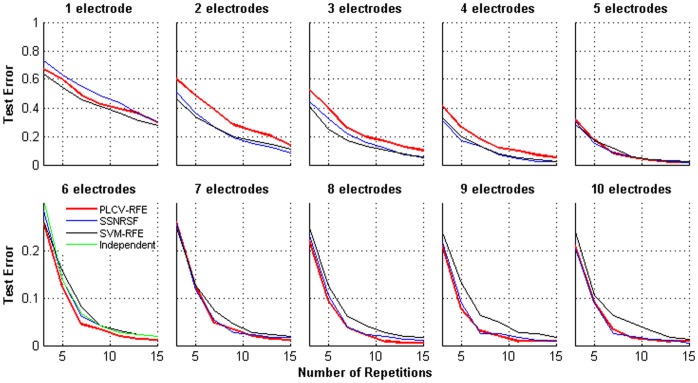
The comparison of character recognition errors. CREs of 1 to 10-channel subsets are shown against the number of repetitions with different methods.

A further comparison is shown in Figure 7. Since a practical BCI system prefers a small electrode set, one of the core objectives of a channel selection method is to discover the fewest electrodes with the least test error. Figure 7 displays the averaged CREs of 1 to 10 best subject-specific electrodes with different methods. A channel subset with six electrodes can be found in many studies on the P300 speller, and has been proven to be able to provide a classification performance as good as other expanded channel sets [20],[21]. Therefore, we make another comparison with the performance of a subject-independent six-channel set whose locations are predefined following previous studies on the P300 speller [21]. Among these four-channel sets, the PLCV-RFE achieves the lowest CRE and three paired t-tests demonstrate significant superiority (in the section of 3 to 15 repetitions, PLCV-RFE versus SSNRSF: *t*(116) = −2.7, *p*-value<0.01, PLCV-RFE versus SVM-RFE: *t*(116) = −2.3, *p*-value<0.05 and PLCV-RFE versus the independent: *t*(116) = −2.9, *p*-value<0.01). In addition, the PLCV-RFE performs significantly superior to others in 8 electrodes (in the section of 3 to 15 repetitions, PLCV-RFE versus SSNRSF: *t*(116) = −2.0, *p*-value<0.05, PLCV-RFE versus SVM-RFE: *t*(116) = −4.1, *p*-value<0.01). However, it is less effective when using only 2 to 4 electrodes.

In this study, there are three feature selection methods compared from the view of performance of channel selection in the P300 speller. These methods are derived from different mathematical ideas. The SVM-RFE is a kind of dependent criterion that has been successfully used in many areas, such as channel selection in motor imagery-based BCI. As the SVM-RFE is an efficient universal algorithm, it can provide satisfactory results in many cases, but may be not the best one such as in this study. The SSNRSF and PLCV-RFE are both independent criteria with global measurements of the EEG signal. The SSNRSF assumes that spelling responses are additive to the spontaneous electroencephalogram and other background artifacts according to the mechanism of the evoked model. The quality of evoked potentials is reflected by signal to signal-plus-noise ratio, which is related to the classification accuracy in the SSNRSF. The aim of using the spatial filter xDWAN is to amplify the evoked energy, while restraining the background noise. However, the PLCV-RFE works with the assumption of phase correlation between spelling responses. The idea is rooted in the oscillatory model which believes that stimuli induce a phase reset of ongoing neural activity. If the phase resetting is an acceptable reason for the generation of ERP just as suggested in [38]–[40], a proper phase measurement approach can reflect abundant information about the EEG evolution with which to select channels. Therefore, the outstanding performance of PLCV-RFE may be an evidence to indirectly support that the oscillatory model is at least a partial reason for the ERP generation in BCI spelling tasks, due to no utilization of energy or SNR information in the PLCV-RFE procedure.

Previous studies have suggested that visual response and cognitive processing are two main neural activities in response to overt target stimulus [46],[47]. Other neurophysiology studies have discovered that at least eight classes of independent components contribute to visual evoked responses [35]. Frenzel *et al* have realized a P300-based BCI system with two parallel communication lines by detecting different brain activities [48]. Therefore, the responded potentials of the P300-speller can be regarded as a mixed effect of many sources, which is consistent with the idea of PLCV-RFE that pursues the new features from different sources to robust channel selection.

From the view of results, the best channel location set is subject-specific for users to control the P300 speller. And especially for patients suffering from central nervous disorders, a measurement of the best channel locations is beneficial and helpful for them to use the BCI system, since the great change of brain structures and functions may influence the normal EEG signal. This paper introduces a novel approach to select channels in P300 speller paradigms. The PLCV-RFE, as a phase measurement based channel selection algorithm, can effectively remove the less important channels without loss of classification accuracy, and shows better performance than other state-of-the-art methods in this study. Thus, phase measurement is effective in channel selection of BCI spelling.

## References

[1] Farland DJ, Wolpaw JR (2011) Brain-computer interfaces for communication and control. Commun ACM. 54: 60–66. 10.1145/1941487.1941506 PMC3188401 21984822

[2] V aughan TM, Heetderks WJ, Trejo LJ, Rymer WZ, Weinrich M, et al (2003) Brain-computer interface technology: a review of the second international meeting. IEEE Trans. Neural Syst. Rehabil. Eng. 11: 94–109. 10.1109/tnsre.2003.814799 12899247

[3] Allison BZ, Wolpaw EW, Wolpaw JR (2007) Brain-computer interface system: progress and prospects. Expert Rev. Med. Devices 4: 463–74. 10.1586/17434440.4.4.463 17605682

[4] Nijboer F, Sellers EW, Mellinger J, Jordan MA, Matuz T, et al (2008) P300-based brain-computer interface for people with amyotrophic lateral sclerosis. Clin. Neurophysiol. 119: 1909–16. 10.1016/j.clinph.2008.03.034 PMC2853977 18571984

[5] Sellers EW, Vaughan TM, Wolpaw JR (2010) A brain-computer interface for long-term independent home use. Amyotroph. Lateral. Scler. 11: 449–55. 10.3109/17482961003777470 20583947

[6] Vaughan TM, McFarland DJ, Schalk G, Sarnacki WA, Krusienski DJ, et al (2006) The Wadsworth BCI research and development program: at home with BCI. IEEE Trans. Neural Syst. Rehabil. Eng. 14: 229–33. 10.1109/TNSRE.2006.875577 16792301

[7] Farwell LA, Donchin E (1988) Talking off the top of your head: toward a mental prosthesis utilizing event-related brain potentials. Electroencephalogr. Clin. Neurophysiol. 70: 510–23. 10.1016/0013-4694(88)90149-6 2461285

[8] Pritchard WS (1981) Psychophysiology of P300. Psychol. Bull. 89: 506–40. 7255627

[9] Mak JN, Arbel Y, Minett JW, McCane LM, Yuksel B, et al (2011) Optimizing the P300-based brain-computer interface: current status, limitations and future directions. J. Neural Eng. 8: 025003. 10.1088/1741-2560/8/2/025003 21436525

[10] Cecotti H, Rivet B, Congedo M, Jutten C, Bertrand O, et al (2011) A robust sensor selection method for P300 brain-computer interfaces. J. Neural Eng. 8: 016001. 10.1088/1741-2560/8/1/016001 21245524

[11] Shih EI, Shoeb AH, Guttag JV (2009) Sensor selection for energy-efficient ambulatory medical monitoring. In Proc. 7th Int. Conf. on Mobile Systems, Applications and Services: 22–25 June, 2009; Krakow. New York: Association for Computing Machinery. 347–58.

[12] Hillyard SA, Kutas M (1983) Electrophysiology of cognitive processing. Annu. Rev. Psychol. 34: 33–61. 10.1146/annurev.ps.34.020183.000341 6338812

[13] Donchin E, Karis D, Bashore TR, Coles MGH, Gratton G (1986) Cognitive Psychophysiology and human information processing. In Coles MGH, Donchin E and Porges SW editors. Psychophysiology: Systems, processes, and applications. New York: Guilford Press. 244–267.

[14] Blankertz B, Müller KR, Curio G, Vaughan TM, Schalk G, et al (2004) The BCI competition 2003: progress and perspectives in detection and discrimination of EEG single trials. IEEE Trans. Biomed. Eng. 51: 1044–51. 10.1109/TBME.2004.826692 15188876

[15] Blankertz B, Müller KR, Krusienski DJ, Schalk G, Wolpaw JR, et al (2006) The BCI competition III: validating alternative approaches to actual BCI problems. IEEE Trans. Neural Syst. Rehabil. Eng. 14: 153–9. 10.1109/TNSRE.2006.875642 16792282

[16] Kaper M, Meinicke P, Grossekathoefer U, Lingner T, Ritter H (2004) BCI competition 2003-data set IIb: support vector machines for the P300 speller paradigm. IEEE Trans. Biomed. Eng. 51: 1073–6. 10.1109/TBME.2004.826698 15188881

[17] Spencer KM, Dien J, Donchin E (2001) Spatiotemporal analysis of the late ERP responses to deviant stimuli. Psychophysiology 38: 343–58. 11347879

[18] Vaughan TM, McFarland DJ, Schalk G, Sellers E, Wolpaw JR (2003) Multichannel data from a brain-computer interface (BCI) speller using a P300 (i.e., oddball) protocol. Soc Neurosci Abs.

[19] Hoffmann U, Vesin JM, Diserens K, Ebrahimi T (2008) An efficient P300-based brain-computer interface for disabled subjects. J. Neurosci. Methods 167: 115–25. 10.1016/j.jneumeth.2007.03.005 17445904

[20] Krusienski DJ, Sellers EW, McFarland DJ, Vaughan TM, Wolpaw JR (2008) Toward enhanced P300 speller performance. J. Neurosci. Methods 167: 15–21. 10.1016/j.jneumeth.2007.07.017 PMC2349091 17822777

[21] Liu Y, Zhou Z, Hu D (2011) Gaze independent brain-computer speller with covert visual search tasks. Clin Neurophysiol. 122: 1127–36. 10.1016/j.clinph.2010.10.049 21163695

[22] Krusienski DJ, Sellers EW, Cabestaing F, Bayoudh S, McFarland DJ, et al (2006) A Comparison of Classification Techniques for the P300 Speller. J. Neural Eng. 3: 299–305. 10.1088/1741-2560/3/4/007 17124334

[23] Rakotomamonjy A, Guigue V (2008) BCI Competition III: Dataset II- Ensemble of SVMs for BCI P300 Speller. IEEE Trans. Biomed. Eng. 55: 1147–54. 10.1109/TBME.2008.915728 18334407

[24] Lal TN, Schröder M, Hinterberger T, Weston J, Bogdan M, et al (2004) Support Vector Channel Selection in BCI. IEEE Trans. Biomed. Eng. 51: 1003–10. 10.1109/TBME.2004.827827 15188871

[25] Schroder M, Bogdan M, Rosenstiel W, Hinterberger T, Birbaumer N (2003) Automated EEG feature selection for brain computer interfaces. In Proceedings of the 1st International IEEE EMBS Conference on Neural Engineering: 20–22 March 2003; Capri. 626–9.

[26] Rivet B, Souloumiac A, Gibert G, Attina V (2008) P300 speller brain-computer interface: enhancement of P300 evoked potential by spatial filters. In Proc. European Signal Processing Conf. (EUSIPCO), Lausanne.

[27] Rivet B, Souloumiac A, Attina V, Gibert G (2009) XDAWN algorithm to enhance evoked potentials: application to brain-computer interface. IEEE Trans. Biomed. Eng. 56: 2035–43. 10.1109/TBME.2009.2012869 19174332

[28] Hillyard SA (1985) Electrophysiology of human selective attention. Trends Neurosci. 8: 400–405.

[29] Vaughan HG, Arezzo JC (1988) The neural basis of event-related potentials. In Picton TW edtor, Human Event-Related Potentials vol. 3. New York: Elsevier Science Publishers. 45–94.

[30] Schroeder CE, Steinschneider M, Javitt DC, Tenke CE, Givre SJ, et al (1995) Localization of ERP generators and identification of underlying neural processes. Electroencephalogr. Clin. Neurophysiol. 44: 55–75. 7649056

[31] Makinen V, May P, Tiitinen H (2004) Transient brain responses predict the temporal dynamics of sound detection in humans. NeuroImage 21: 701–706. 14980572 10.1016/j.neuroimage.2003.10.009

[32] Sayers BM, Beagley HA, Hensall WR (1974) The mechanism of auditory evoked EEG responses. Nature 247: 481–483. 4818547 10.1038/247481a0

[33] Basar E (1980) EEG-Brain Dynamics: Relation Between EEG and Brain Evoked Potentials. New York, Elsevier.

[34] Brandt ME, Jansen BH, Carbonari JP (1991) Pre-stimulus spectral EEG patterns and the visual evoked response. Electroencephalogr. Clin. Neurophysiol. 80: 16–20. 10.1016/0168-5597(91)90037-x 1703944

[35] Makeig S, Westerfield M, Jung TP, Enghoff S, Townsend J, et al (2002) Dynamic Brain Sources of Visual Evoked Responses. Science 295: 690–4. 11809976 10.1126/science.1066168

[36] Kruglikov SY, Schiff SJ (2003) Interplay of electroencephalogram phase and auditory-evoked neural activity. J. Neurosci. 23: 10122–10127. 10.1523/JNEUROSCI.23-31-10122.2003 PMC6740863 14602828

[37] Sauseng P, Klimesch W (2008) What does phase information of oscillatory brain activity tell us about cognitive processes? Neurosci Biobehav Rev. 32: 1001–13. 10.1016/j.neubiorev.2008.03.014 18499256

[38] Sauseng P, Klimesch W, Gruber WR, Hanslmayr S, Freunberger R, et al (2007) Are event-related potential components generated by phase resetting of brain oscillations? A critical discussion. Neuroscience 146: 1435–44. 17459593 10.1016/j.neuroscience.2007.03.014

[39] Fuentemilla L, Marco-Pallarés J, Grau C (2006) Modulation of spectral power and of phase resetting of EEG contributes differentially to the generation of auditory event-related potentials. Neuroimage 30: 909–16. 16376575 10.1016/j.neuroimage.2005.10.036

[40] Hanslmayr S, Klimesch W, Sauseng P, Gruber W, Doppelmayr M, et al (2007) Alpha Phase Reset Contributes to the Generation of ERPs. Cereb Cortex 17: 1–8. 16452640 10.1093/cercor/bhj129

[41] Le Van Quyen M (2003) Disentangling the dynamic core: A research program for a neurodynamics at the large-scale. Biol. Res. 36: 67–88. 10.4067/s0716-97602003000100006 12795207

[42] Geng T, Gan JQ, Dyson M, Tsui CS, Sepulveda F (2008) A novel design of 4-class BCI using two binary classifiers and parallel mental tasks. Comput. Intell. Neurosci. 437306. 10.1155/2008/437306 PMC2435224 18584040

[43] Varela F, Lachaux JP, Rodriguez E, Martinerie J (2001) The brainweb: phase synchronization and large-scale integration. Nat. Rev. Neurosci. 2: 229–39. 10.1038/35067550 11283746

[44] Brunner C, Scherer R, Graimann B, Supp G, Pfurtscheller G (2006) Online control of a brain-computer interface using phase synchronization. IEEE Trans. Biomed. Eng. 53: 2501–6. 10.1109/TBME.2006.881775 17153207

[45] Lachaux JP, Rodriguez E, Martinerie J, Varela FJ (1999) Measuring phase synchrony in brain signals. Hum. Brain Mapp. 8: 194–208. 10.1002/(SICI)1097-0193(1999)8:4<194::AID-HBM4>3.0.CO;2-C PMC6873296 10619414

[46] Brunner P, Joshi S, Briskin S, Wolpaw JR, Bischof H, et al (2010) Does the ‘P300’ speller depend on eye gaze? J. Neural Eng. 7: 056013. 10.1088/1741-2560/7/5/056013 PMC2992970 20858924

[47] Treder MS, Blankertz B (2010) (C)overt attention and visual speller design in an ERP-based brain-computer interface. Behav. Brain Funct. 6: 28. 10.1186/1744-9081-6-28 PMC2904265 20509913

[48] Frenzel S, Neubert E, Bandt C (2011) Two communication lines in a 3 × 3 matrix speller. J Neural Eng. 8: 036021. 10.1088/1741-2560/8/3/036021 21555846

